# The challenges of access to innovative medicines with limited evidence in the European Union

**DOI:** 10.3389/fphar.2023.1215431

**Published:** 2023-08-31

**Authors:** Antonio Vallano, Caridad Pontes, Antònia Agustí

**Affiliations:** ^1^ Medicines Department, Catalan Healthcare Service, Barcelona, Spain; ^2^ Department of Pharmacology, Therapeutics and Toxicology, Universitat Autònoma de Barcelona, Barcelona, Spain; ^3^ Healthcare Management of Hospitals, Catalan Institute of Health, Barcelona, Spain; ^4^ Digitalization for the Sustainability of the Healthcare System DS3-IDIBEL, L’Hospitalet de Llobregat, Spain; ^5^ Clinical Pharmacology Service, Vall d’Hebron University Hospital, Barcelona, Spain

**Keywords:** drug approval, drug costs, orphan drug, antineoplastic agents, European Union

## Abstract

The European Medicines Agency (EMA) fosters access to innovative medicines through accelerated procedures and flexibility in the authorization requirements for diseases with unmet medical needs, such as many rare diseases as well as oncological diseases. However, the resulting increase of medicines being marketed with conditional authorizations and in exceptional circumstances has lead to higher clinical uncertainty about their efficacy and safety than when the standard authorizations are applied. This uncertainty has significant implications for clinical practice and the negotiation of pricing and reimbursement, particularly as high prices are based on assumptions of high value, supported by regulatory prioritization. The burden of clinical development is often shifted towards public healthcare systems, resulting in increased spending budgets and opportunity costs. Effective management of uncertainty, through appropriate testing and evaluation, and fair reflection of costs and risks in prices, is crucial. However, it is important not to sacrifice essential elements of evidence-based healthcare for the sake of access to new treatments. Balancing sensitive and rational access to new treatments, ensuring their safety, efficacy, and affordability to healthcare systems requires thoughtful decision-making. Ultimately, a responsible approach to timely access to innovative medicines that balances the needs of patients with healthcare systems’ concerns is necessary. This approach emphasizes the importance of evidence-based decision-making and fair pricing and reimbursement.

## 1 Introduction

In recent years, there have been significant technological advances in biomedical research that have been quickly translated into clinical practice (Zeggini et al., 2019; Tsimberidou et al., 2020). The pharmaceutical industry is shifting its focus from traditional research and development programs targeting common diseases to a new approach of discovering treatments for rare and hard-to-treat illnesses with unmet medical needs (Attwood et al., 2018). However, these advances also come with a significant increase in healthcare costs (Keehan et al., 2015).

The European Medicines Agency (EMA) plays a significant role in evaluating pharmacological innovations and issuing opinion for their commercialization in the European Union (EU) countries (European Medicines Agency, 2020a). The European Commission then ultimately authorizes the marketing of these medicines in the EU (EUR-Lex, 2004). However, the decision on the pricing and financing of these medications with public funds is a competence of the individual member states (Antoñanzas et al., 2005; Löblová, 2021; Vončina et al., 2021). Finally, regional or local governments, health centers, and healthcare professionals are responsible for deciding which medications to prioritize for certain patients or circumstances in a domestic context.

There is a demand that the process of access to innovative medicines should be faster, and patients should have timely access to new and innovative medicines (Annemans et al., 2011; Baird et al., 2014; Panteli and Edwards, 2018). For life-threatening or debilitating diseases that have limited or no treatment options, access to new drugs can provide relief and improve the quantity and quality of life for existing patients with ominous prognosis. However, many healthcare stakeholders claim that numerous patients with life-threatening or debilitating diseases still do not have access to new and innovative medicines (Baird, et al., 2014; Panteli and Edwards, 2018; Horgan, et al., 2022).

The EMA has acknowledged the existence of unmet medical needs and have established laws and regulations aimed at expediting the development and approval of drugs to address these specific diseases (EUR-Lex, 2004). A group of experts with representatives from the rare disease community, researchers, patient advocates, investors, and pharmaceutical companies has proposed several measures to promote rare disease medicine, including a faster regulatory process (Aartsma-Rus et al., 2021). However, when it comes to regulatory processes enabling quick access to new medicines, it entails accepting a higher level of uncertainty during the approval stage. This has sparked ongoing discussions regarding the most suitable trade-off between speed and the evidence required for the development of new medicines.

In this article we will delves into the complex subject of medications authorized with limited clinical evidence. There are several reasons for granting access to medicines with limited evidence (Table 1). We aim to examine various scenarios encountered in clinical research, including trials conducted without a control group, studies involving a restricted number of participants and limited available information, utilization of surrogate endpoints, accelerated authorizations based on promising initial results pending confirmation from more robust data, as well as conditional authorizations granted under exceptional circumstances. Furthermore, our attention is directed towards the intricate challenges that arise during the decision-making process concerning the funding of these medications. This is particularly noteworthy due to the limited clinical evidence and frequently elevated costs associated with such treatments. Therefore, a thorough evaluation of resource allocation and an assessment of the value and cost-effectiveness associated with these interventions are needed.

**TABLE 1:** Justification for access to medicines with limited evidence.

• Diseases with limited treatment options
• Urgency of treatment in life-threatening or debilitating diseases
• Opportunity costs of patients at high unmet medical needs
• Difficulties and high costs of research in small populations at high need
• Fast dissemination of early evidences of potentially breakthrough therapies
• Regulatory recognition of unmet medical needs
• Social demand of access to innovation

## 2 Actions to accelerate regulatory access to innovative medicines

Medicine regulation by the EMA aims to ensure that only medicines with a favorable balance of benefits and risks are authorized for marketing. This requires the assessment of three criteria: quality, efficacy, and safety (European Medicines Agency, 2020a). However, conducting the necessary studies to evaluate these criteria can be costly and time-consuming. Incentives have been put in place to encourage research and innovation in areas with high unmet medical need, which can result in a flexibility of regulatory requirements and shortened assessment timelines to avoid delays in access to treatment, especially for serious and urgent illnesses. Therefore, in some cases the regulation procedure offers incentives and is faster and more flexible.

The European Union incentivizes the development of medicines that are intended to treat small patient populations, assuming that the development of these types of medicines may not be financially viable under normal market conditions. The EMA’s Committee for Orphan Medicinal Products (COMP) grants orphan designation to medicines that treat life-threatening diseases with a low prevalence in the EU and offer significant benefits over existing treatments or fill a gap where no satisfactory treatments exist. Orphan drug designation recognizes that the drug is addressing a relevant unmet need, and offers several incentives, including reduced or waived fees, protocol assistance (scientific advice specific to orphan drugs), and 10 years market exclusivity in the EU (EUR-Lex, 2000; European Medicines Agency, 2022a). Orphan medicinal products (OMP) are qualified as such after receiving a positive opinion from the Committee for Medicinal Products for Human Use (CHMP) (European Medicines Agency, 2022a).

Accelerated review processes have been developed to reduce the time required by the EMA to review a marketing authorization application for medicines that are considered important therapeutic innovations and are of great public health interest. This expedited review process reduces the review procedure time from 210 days to 150 days, if the applicant provides good cause for an expedited review (European Medicines Agency, 2021a).

The PRIME (PRIority MEdicines) program is an initiative developed by the EMA to improve and accelerate the evaluation and approval process of medicinal products aimed to treat serious and life-threatening conditions with unmet medical needs. The program offers ongoing assistance for the advancement of qualified medications, which have been chosen based on their potential to provide substantial therapeutic benefits compared to current treatments or to benefit patients who lack treatment options altogether. The primary objective is to streamline the medicine development process and expedite access to these innovative treatments (European Medicines Agency, 2023a). The PRIME program provides early and proactive support to medicine developers to generate robust data on the benefits and risks of a drug, and to accelerate the assessment of applications for medicine approvals through early interaction and dialogue with regulators. PRIME allows applicants to receive confirmation during the clinical development phase on whether their drug may be eligible for accelerated assessment (European Medicines Agency, 2023a).

A comprehensive review of the PRIME scheme’s experience since inception and up to June 2021 has been carried out (European Medicines Agency, 2018a; European Medicines Agency, 2022b). The monthly average of PRIME applications in the period was 6.1, with a total of 384 requests of which 25% (N = 95) being granted. Oncology products made up the majority of applications (29%), while advanced therapy medicinal products (ATMPs) had the highest success rate (46%). Orphan-designated products made up 42% of PRIME eligibility applications, and 56% of PRIME products granted eligibility had an orphan designation. Medicines with a PRIME designation more often have conditional authorizations than medicines without this designation (European Medicines Agency, 2022b).

**FIGURE 1:**
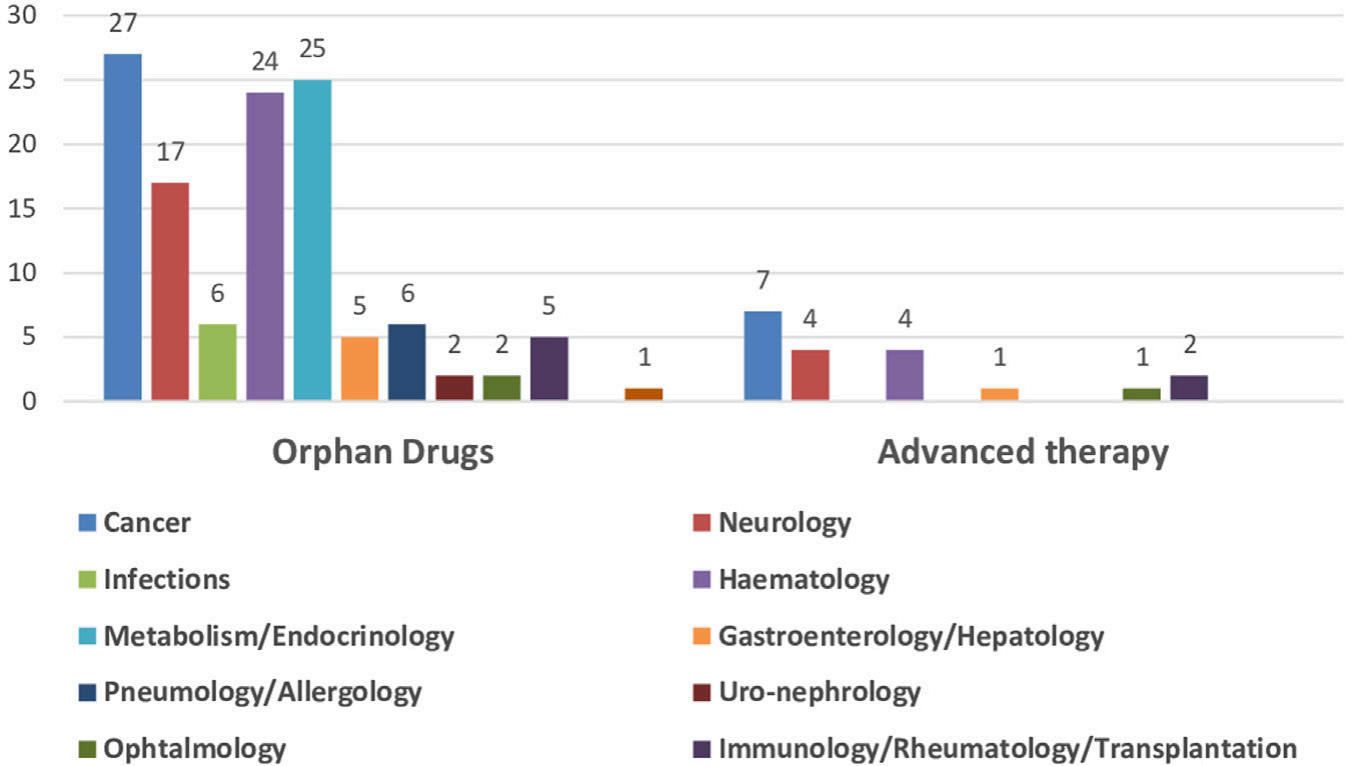
Number of orphan drugs and advanced therapies authorized by CHMP to treat diseases in different therapeutic areas, 2016–2021.

**FIGURE 2:**
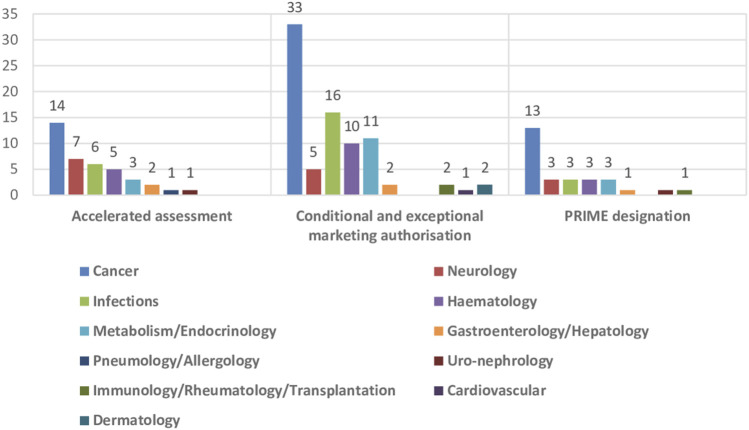
Number of accelerated assessments, conditional and exceptional marketing authorisations, and PRIME designations for medicines with positive opinion from the CHMP for treating diseases in different therapeutic areas, 2015–2021.

The impact of the regulatory procedures mentioned above has been a progressive increase in the authorization of medicines with orphan and advanced therapies designations (European Commission, 2020; Technopolis Group, 2020) (Table 2). In addition, conditional marketing authorizations and marketing authorizations under exceptional circumstances have also increased, as well as, PRIME designation and accelerated authorization procedures (Table 2) (European Medicines Agency, 2016; European Medicines Agency, 2017; European Medicines Agency, 2018b; European Medicines Agency, 2019; European Medicines Agency, 2020b; European Medicines Agency, 2021b; European Medicines Agency, 2022c; European Medicines Agency, 2023b). The number of orphan drugs and advanced therapies with positive opinion of CHMP to treat diseases in different Therapeutic Areas from 2016 to 2021 is shown in Figure 1. The number of accelerated assessments, conditional and exceptional marketing authorizations, and PRIME designations for medicines with positive opinion from the CHMP for treating diseases in different therapeutic areas from 2015–2021 is shown in Figure 2. Oncology is the therapeutic area with more orphan drugs, advanced therapies, accelerated assessments, conditional and exceptional marketing authorizations, and PRIME designations.

**TABLE 2:** Medicines with positive opinions from the European Medicines Agency’s Committee for Medicinal Products for Human Use (CHMP) from 2015 to 2022.

	2015 (n = 92)	2016 (n = 81)	2017 (n = 92)	2018 (n = 84)	2019 (n = 66)	2020 (n = 97)	2021 (n = 92)	2022 (n = 89)	Total (n = 693)
News active ingredients	39	27	35	42	30	39	54	41	307
Orphan medicines	18	16	19	21	7	22	19	21	143
ATMPs	1	2	2	3	1	3	2	6	20
Accelerated procedures of authorization	5	7	7	4	3	5	3	5	39
PRIME program				3	3	8	6	8	28
Type of marketing authorization									
Standard	87	72	87	80	57	79	75	75	612
Conditional	3	8	3	1	8	13	13	9	58
Exceptional circumstances	3	1	2	3	1	5	4	5	24

ATMPs, Advanced therapy medicinal products.

It must be emphasized that currently the European Commission is proposing a review of European Union pharmaceutical legislation, consisting of a new Directive and a Regulation to simplify and replace the previous legislation. The new legislation will promote innovation in the development of new medicines by speeding up the authorization process with simplified procedures, offering tailored scientific support and advice for innovative products, and providing special incentives for rare diseases. The reform aims to provide all patients in the EU with timely and equitable access to safe and effective medicines, and offering incentives for the development of innovative medicines addressing unmet medical needs (European Commission, 2023).

## 3 Uncertainty and consequences of access to innovative medicines with limited evidence

### 3.1 Uncertainty of benefit at authorization and during the post-marketing period

However, rapid access to new medicines from the regulatory side means that in most cases, more uncertainty is accepted at the time of approval, and there is still debate about the optimal balance between speed and evidence for developing new medicines. A review of oncology medicines approved by the EMA from 2015 to 2020 found that most medicines were approved for marketing based on surrogate outcomes, without evidence of improved overall survival (OS) or quality of life benefits (Falcone et al., 2022).

Moreover, there is concern on the actual magnitude and relevance of the clinical benefit, and balance with added toxicities of newly authorized products. An analysis of OS data of new cancer medicines approved by the US Food and Drug Administration (FDA) and the EMA between 2003 and 2013 found that only 43% of the drugs showed an increase of at least 3 months, 11% showed less than 3 months, and 30% showed no improvement in OS. The average increase in OS was of 3.4 months, and the majority of the new cancer medicines were also associated with increased toxicity (Vega et al., 2017). The analysis of 38 cancer medicines for solid tumors approved by the EMA between 2011 and 2016 found that the results of 89% out of the 70 supporting pivotal trials did not meet the threshold of clinical relevance on the ESMO-MCBS scale, suggesting that the clinical benefit of these drugs may be questionable (Grössmann et al., 2017).

To note, clinical trials are increasingly adopting methodologies that allow early interruption led by interim analysis, if supporting positive results. However, early trial interruption may led to overestimation of effects, especially if decisions are not taken blinded to treatment groups, or are based on uncontrolled designs (Montori et al., 2005; Bassler et al., 2010). Often, sponsors do not adequately report on the decision to stop, and large treatment effects are show that are unlikely to be confirmed later on, especially when the number of events is small. A study of clinical trials that were stopped early for benefit found that these trials typically included 63% of the planned sample size and were stopped after a median of 13 months of follow-up, with an intermediate interim analysis and a median of 66 patients. The trials did not report at least one of the following characteristics: planned sample size, interim analysis, whether a stopping rule informed the decision, or an adjusted analysis accounting for interim follow-up and truncation. Trials with fewer events produced larger treatment effects, thus suggesting that the results of these early-stopping trials may be frail and potentially biased, and should be regarded with high caution (Montori et al., 2005; Walter et al., 2019; Liu and Garrison, 2022).

On top of that, the evidence on new drugs that are addressed to small populations, such as OMPs and ATPMs, may also be flawed by the use of weak methodological approaches. A review of the European Public Assessment Reports (EPARs) of 125 OMPs approved by the EMA between 1999 and 2014 found that one third of the trials did not include a control arm, one third did not use randomization, half of the trials were open-label, and 75% used intermediate or surrogate outcomes as the primary endpoint. The size of the population exposed at the time of OMPs approval was smaller than needed to classify adverse reactions as clinically relevant, and 10% of the OMPs were approved despite the results of the pivotal trials being negative (Pontes et al., 2018). This suggests that the regulatory evidence supporting OMPs approval had significant uncertainties, including weak protection against bias, substantial use of inappropriate study designs, reliance on intermediate outcomes, lack of prioritization, and insufficient safety data to accurately quantify risks.

Currently, some ATMPs have already been commercialized. A review of pivotal clinical trials (CTs) supporting the approval of ATMPs by EMA found that their approval was mainly based on small CTs, single-arm, no control group, compared to historical controls, and using surrogate outcomes as the primary endpoint (Iglesias-Lopez et al., 2021a). Additionally, in an analysis of the ATMPs approved by EMA and FDA, many of ATMPs had an orphan drug designation, expedited program designation, quick decision on marketing authorization, and non-standard marketing authorization (Iglesias-Lopez et al., 2021b). There are various health and economic adverse consequences with the marketing of medications with limited clinical evidence. Since these medicines are still in the early stages of development, the safety and efficacy of the medicine may not be well established, and thus one of the primary risks of their market access is the lack of sufficiently robust safety and efficacy data for these medicines. This may risk serious side effects and lack of meaningful benefit to the patient. While additional information will be accrued on the efficacy and safety of the medicines in the early marketing period, clinical trials may become unfeasible for recruitment once the product is available, and observational data often does not provide further relevant and robust evidence, so that clinical uncertainty may not be resolved.

From 2009 to 2013, the EMA approved the use of 48 anticancer medicines for 68 indications. In 12% of the indications pivot trials had a unique arm study, in 35%, OS data were not available, and in those that were available, the median overall survival benefit was 2.7 months (range 1–5.8 months). Quality of life data only were available in 10% of cases. In post-marketing follow-up data only 3 of 44 indications that initially had no overall survival data showed an overall survival gain or benefit in post-marketing outcomes. Median follow-up after authorization was 5.4 years (range, 3.3–8.1 years). Of the 23 drugs with an ESMO score, 12 (52%) had a score indicating non-significant improvement. For about half (33; 49%) the post-marketing benefit was still uncertain. Therefore, about 50% of medicines authorized for licensed oncology indications remained uncertain after an average of 5.4 years after approval (Davis et al., 2017).

Conditional marketing authorizations should be reversible if benefit assumptions are not met, but in clinical practice, they barely are. For instance, in the case of ataluren, the conditional marketing authorization by EMA was issued without conclusive evidence on efficacy in pivotal clinical trials, but based on contextual reasons and a reasonably safety profile (Haas et al., 2015). Subsequent clinical trials failed to conclude efficacy, but the medicine is still commercialised with annual treatment costs over €200,000 per year (McDonald et al., 2017). Olaratumab received an accelerated and conditional approval based on exceptional efficacy in a single phase 2 trial, awaiting the results of a phase III clinical trial, and rapidly taken up into clinical practice. However, the phase 3 trial failed to conclude efficacy and was withdrawn by the company, although thousands of patients had already been treated in the EU with a cost greater than thirty million euros (Pontes et al., 2020).

### 3.2 Uncertainty in pricing and reimbursement process

Pricing and reimbursement (P&R) decisions for innovative medicines are a complex and challenging task for health systems, as they balance the need to provide access to new treatments with the need to control costs and ensure long-term sustainability. The P&R process involves evaluation of the clinical and economic value of new medicines, considering factors such as clinical efficacy, safety, cost-effectiveness, and budget impact. It also requires taking into account the perspectives of various stakeholders, including patients, pharmaceutical companies, healthcare providers, payers, and policymakers. When there is a great unmet medical need and no therapeutic alternatives available, the decision making process becomes even more challenging. The perception of high value for accelerated, conditional, or exceptional authorizations can lead to high expectations, associated with high prices, from agents of interest and social pressure for hard bargaining. In these cases, there are no competitors, and therefore no comparative data to go on.

Companies are unwilling to set low prices for their products, tend to overestimate the cost-effectiveness of their therapies, and claim theoretical prices that are expected to return costs of manufacturing, R&D investments and reward value of innovation (Saluja et al., 2021). However, R&D costs are not transparent and traceable enough, there are no clear rules on how to consider the amount of effort done up to the P&R decision (especially in early approvals) nor on how different countries must bear and share the burden of such investment returns. Consequently, a variation in pricing, funding decisions, and time to reimbursement for innovative medicines, which encompasses OMPs, ATPMs, and anticancer drugs, across European countries has been described (Martinalbo et al., 2016; Szegedi et al., 2018; Cufer et al., 2020; Iglesias-Lopez et al., 2022; Post et al., 2023). This fuels disparities in patient access to these medicines throughout the diverse European countries. It is worth highlighting that indications for use of the new innovative medicines, such as OMPs, trend to be progressively smaller, while their relative spending has steppedly increased over 20 years across European countries, and the cost per patient is progressively higher (Mestre-Ferrandiz et al., 2019). Medicines with costs per patient exceeding 1 million euros have recently been marketed (Nuijten, 2022) The proliferation of very expensive drugs has sparked debate about their sustainability and affordability (Kang et al., 2021).

## 4 Discussion

Access to innovative medicines for patients with rare diseases and unmet medical needs is crucial. However, the uncertainties inherent in developing those medicines with a limited evidence pose significant challenges to traditional health technology assessment, and P&R processes.

Firstly and foremost, it is vital to enhance the scientific evidence of those medications. Classic confirmatory clinical trial designs with randomization and control groups, with the best available treatment option, should be the best option, as longas it is feasible (Hulley et al., 2013). This approach ensures rigorous evaluation of treatment efficacy and safety. In addition, it is important to evaluate variables that are clinically relevant, rather than surrogate variables, and demonstrate benefits that are of clinical relevance. This means focusing on outcomes that directly impact patients’ health and wellbeing. Broadening eligibility criteria and avoiding unnecessary exclusions can help to increase the number of included patients in clinical trials, particularly when addressing rare diseases. However, within the realm of rare diseases, where patient populations tend to exhibit a notable heterogeneity, the mere expansion of participant numbers could potentially complicate the interpretation of trial outcomes. This complication might give rise to challenges in pinpointing the specific subgroups that derive benefits from treatments, owing to the inadvertent inclusion of patients with disparate phenotypes. If the traditional design is not feasible, alternative designs such as adaptive designs, and trial designs that aim to gather the maximum amount of useful data from a reduced number of patients could be considered (Pallmann et al., 2018; Subbiah, 2023). Post-marketing real world data has been put forth as a potential surrogate in the absence of good evidence from clinical trials (Swift et al., 2018). However, pragmatic post-marketing research produces a less robust evidence than pre-marketing experimental studies (Makady et al., 2019a).

Secondly, payers may have doubts about the effectiveness, safety, and therapeutic value of a treatment that has not been fully confirmed because they need accurate information to decide P&R of these innovative medicines (Simoens, 2011). There is a concern that payers and society may be burdened with the costs of unproven yet expensive treatments. Healthcare stakeholders should take a comprehensive approach to assess decision-making on access to innovative medicines with limited evidence. This may include restrictive access decisions for conditionally approved products through requesting robust evidence based on well-designed clinical trials able to evaluate both relevant clinical and non-clinical outcomes, in order to ensure guarantees of efficacy and safety of those products and economic sustainability at the population level (Lau and Dranitsaris, 2022).

The regulatory approval process has undergone meticulous review and adaptation to facilitate access of innovative medicines. Similarly, there seems to be a need to revisit the P&R system, through a transparent and evidence-based approach, as well as an effective price regulation, that is able to manage the greater amount of uncertainty resulting from regulatory measures to accelerate access of innovation (World Health Organization Regional office for Europe, 2018).

Fixing a price on a population level, as well as a spending cap per patient, in an uncertain setting should not result in premium prices based on expectations, but on prices that are proportional to its value at the time of P&R, considering the magnitude of benefit but also the strength and different levels of the evidence supporting it. If the evidence for a medicine’s efficacy and safety is weak, the price should also be lower, regardless of other factors, at least until the expectations can be robustly confirmed.

A strategy often applied to manage clinical uncertainty of expensive medicines aimed to small populations are risk-sharing agreements or managed access agreements (MAA) (Bouvy et al., 2018; Dabbous et al., 2020). Thus, when weak clinical evidence, and value and economic uncertainties derive from a large budget impact, an option is measuring outcomes in clinical practice, and linking actual effectiveness to sharing of financial risks. The collection of additional data after conditional authorization aids to confirm that the benefits outweigh the risks, and to ensure that the medicine is able to meet the needs of the population. In this way, risk-sharing arrangements may balance the need to provide rapid access to potentially beneficial medicines with the need to circumscribe uncertainty, obtaining the best value for money and ensuring affordability (Dabbous et al., 2020). Nevertheless, MAA that require collecting additional data by stakeholders (companies and healthcare professionals) may result in biases in support of access, led by conflicts of financial and clinical interests, respectively.

Curiously, the introduction of a medical product to the market with substantial uncertainty, does not inherently lead to the implementation of performance-based agreements. Between 2006 and 2016, managed entry agreements based on clinical outcomes were not commonly used for products that had a conditional marketing authorization or those that were authorized under exceptional circumstances. Of the 48 products that received marketing authorization under exceptional or conditional circumstances in recent years, only a few were found to have managed entry agreements involving the collection of additional data. The complexity of collecting outcomes data in clinical practice led stakeholders to refrain from utilizing MAA approaches (Bouvy et al., 2018). Besides, risk-sharing agreements can be challenging to implement due to their logistical complexities and the resources required, but also, may not be able to meet their goal of clearing uncertainty. A review of conditional financing agreements in the Netherlands (2006–2012) showed that, in 41% of cases, the data on effectiveness obtained were insufficient to draw conclusions, in 50% additional conditions were required, and in 17% cases there were reasons that advised to suspend reimbursement, but this was unfeasible to implement (Makady et al., 2019b).

Collaboration and early dialogue between stakeholders, including patients, is also crucial to manage expectations and to ensure that access mechanisms are transparent and appropriate (Simoens et al., 2022). The new scenario of accepted uncertainty in some relevant therapeutic areas, such as oncology and orphan diseases, will require further innovative approaches that account for such uncertainty in quantifying therapeutic added value and price. Thus, European countries have adopted different mechanisms for addressing these challenges in oncology. These include approaches aimed directly at the issue, such as multi-year-multi-indication agreements, flexible access agreements for new indications with clinical uncertainty, development of a new agreement for each new indication, and immediate access for new indications and bundled assessments. It is important that policymakers, payers and manufacturers engage in early discussions and are willing to find new solutions to manage appropriately decision on access to innovative medicines (Lawlor et al., 2021).

The Oslo Medicines Initiative (OMI) is a collaborative effort between the WHO Regional Office for Europe, the Norwegian Ministry of Health and Care Services, and the Norwegian Medicines Agency. The OMI aims to provide a neutral platform for the public and the private sectors to jointly outline a vision for equitable and sustainable access to and affordability of effective, novel and high-priced medicines, OMI in a technical report summarizes existing policy options for payers that support innovation and access to medicines in the WHO European Region. It identifies various tools, such as early assessment schemes, managed entry agreements, and innovation funds in 48 countries. The report describes methods for generating evidence and manage access to innovation, such as value-based pricing, pooled procurement, and subscription fee-based procurement. It also acknowledges potential limitations of the identified policies, such as financial sustainability of healthcare systems and trade-offs between incentivizing innovation and principles of evidence generation, transparency, and budget impact (Vogler, 2022).

The health and economic impact of making decisions on access to innovative medicines with limited evidence are significant, and it is essential communicating these issues more effectively to the public. Medicines regulation is designed to protect public health, and the requirement of robust evidence is an ethical obligation to ensure that new treatments are safe and effective. The decision-making process on the price and financing of these innovative medicines must be transparent and based on efficacy, efficiency and affordability, to ensure best use of resources and health system sustainability. Both are safety measures aimed to improve the good for the most, acting as filters rather than barriers, and it would be desirable to ensure that this is perceived as such by the public. Filters are a necessary step to ensure that innovative medicines are safe and effective and that they provide value for money, rather than being bureaucratic obstacles to access. A better communication can help to build a more informed and engaged society, trusting and empowering bodies in charge of veiling for public interests, and ultimately improve public health outcomes.

## 5 Conclusion

In summary, while the EU regulatory process for access to medicines with limited evidence demonstrates flexibility in addressing rare diseases and unmet medical needs, it also introduces substantial clinical uncertainty for public system payers regarding the efficacy and safety of marketed medicines. The consequence of this is uncertainty about the therapeutic place of these drugs in clinical practice, difficulties in making decisions about the price and financing of medicines, and increased budgets for spending on drugs with little evidence.

Based on the above concepts, it is recommended to ensure a balance between flexibility in order to facilitate access to medicines for rare diseases and unmet medical needs, and the need for rigorous clinical research to provide evidence of safety and efficacy at reasonable and affordable prices in order to make the health system sustainable. This could involve implementing well designed clinical trial and gathering post-marketing real world data for innovative medicines granted accelerated authorization, as well as the implementation of a balanced P&R system, through a transparent price regulation, evidence-based approach withprices proportional to the strength and level of evidence and value-based pricing or managed access agreements.

Lastly, it is crucial to communicate the reasoning behind regulatory and financing decisions in a balanced manner. Presently, when regulatory and financing entities seek substantial evidence to guarantee the efficacy, safety, and efficiency of new innovative treatments, the messaging conveyed by media to healthcare professionals and the broader society often frames it as “barriers to innovation”. While there is always area for improvement in procedural efficiency, it is important to visualize the role of public administration bodies in pursuing the best interest for the most. An excessive simplification of messages may push political decisions on access in absence of guarantees on efficacy and safety of those innovative medicines that eventually could be against the general interest. New treatments must be made available to patients as soon as possible, but in a safe, efficient and responsible way. Being fast should not mean rushing but being more responsive. In the fast-paced world of modern medicine, it is easy to get caught up in the race for speed and efficiency. However, true progress lies in balancing speed with accuracy, and we must strive to move forward quickly without sacrificing the essential elements of patient care.
